# Butyrate inhibits the mitochondrial complex Ι to mediate mitochondria-dependent apoptosis of cervical cancer cells

**DOI:** 10.1186/s12906-023-04043-3

**Published:** 2023-06-27

**Authors:** Ke Zhang, Xiawei Ji, Zhengyang Song, Wenjing Song, Qunjia Huang, Tiantian Yu, Dibang Shi, Fangyan Wang, Xiangyang Xue, Junping Guo

**Affiliations:** 1 Department of Pathophysiology, School of Basic Medical Science, Wenzhou Medical University, Wenzhou, 325000 China; 2 Department of Gastroenterology, The Second Affiliated Hospital, Yuying Children’s Hospital of Wenzhou Medical University, Wenzhou, 325000 China; 3 Wenzhou Collaborative Innovation Center of Gastrointestinal Cancer in Basic Research and Precision Medicine, Wenzhou Key Laboratory of Cancer-related Pathogens and Immunity, Department of Microbiology and Immunology, Institute of Molecular Virology and Immunology, School of Basic Medical Sciences, Wenzhou Medical University, Wenzhou, 325000 China; 4 Wuyunshan Hospital of Hangzhou, Health Promotion and Research Institute of Hangzhou, Hangzhou, 310000 China

**Keywords:** Butyrate, Cervical cancer, Mitochondria, Apoptosis, Mitochondrial complex Ι

## Abstract

**Background:**

Cervical cancer (CC) is a common gynecological malignancy with high morbidity worldwide. Butyrate, a short-chain fatty acid produced by intestinal flora, has been reported to inhibit cervical carcinogenesis. This study aimed to investigate the pro-apoptotic effects of butyrate on CC and the underlying mechanisms.

**Methods:**

Human HeLa and Ca Ski cells were used in this study. Cell proliferation, cell migration and invasion were detected by CCK-8 and EdU staining, transwell and wound healing assay, respectively. Cell cycle, mitochondrial membrane potential and apoptosis were evaluated by flow cytometry. Western blot and RT-qPCR were carried out to examine the related genes and proteins to the mitochondrial complex Ι and apoptosis. Metabolite changes were analyzed by energy metabolomics and assay kits. The association between G protein-coupled receptor 41, 43, 109a and CC prognosis was analyzed using data from The Cancer Genome Atlas (TCGA).

**Results:**

CCK-8 results showed significant inhibition of CC cell proliferation induced by butyrate treatment, which was confirmed by EdU staining and cell cycle detection. Data from the transwell and wound healing assay revealed that CC cell migration was dramatically reduced following butyrate treatment. Additionally, invasiveness was also decreased by butyrate. Western blot analysis showed that cleaved Caspase 3 and cleaved PARP, the enforcers of apoptosis, were increased by butyrate treatment. The results of Annexin V/PI staining and TUNEL also showed an increase in butyrate-induced apoptotic cells. Expression of Cytochrome C (Cytc), Caspase 9, Bax, but not Caspase 12 or 8, were up-regulated under butyrate exposure. Mechanistically, the decrease in mitochondrial NADH and NAD + levels after treatment with butyrate was observed by energy metabolomics and the NAD+/NADH Assay Kit, similar to the effects of the complex Ι inhibitor rotenone. Western blot results also demonstrated that the constituent proteins of mitochondrial complex Ι were reduced by butyrate. Furthermore, mitochondria-dependent apoptosis has been shown to be initiated by inhibition of the complex Ι.

**Conclusion:**

Collectively, our results revealed that butyrate inhibited the proliferation, migration and invasion of CC cells, and induced mitochondrial-dependent apoptosis by inhibiting mitochondrial complex Ι.

**Supplementary Information:**

The online version contains supplementary material available at 10.1186/s12906-023-04043-3.

## Introduction

Cervical cancer (CC) is the fourth most common cancer among women worldwide, with 570,000 new cases and 311,000 deaths each year, seriously threatening the life and health of female patients [1]. However, the limited therapeutic effects of current treatments based on incomplete knowledge of its pathogenesis have resulted in the low 5-year survival rate of CC patients [2]. Although human papillomavirus (HPV) is a well-established cause of CC, other factors such as vaginal microbiota likely play an important role [3, 4]. Studies have shown that decreased abundance of *Lactobacillus* and increased *anaerobes* were associated with increased CC severity [5, 6].

Evidences supports that *lactic acid bacteria* exert an anti-cancer effect through several mechanisms, including the production of antitumor metabolites such as lactate and short-chain fatty acids (SCFAs) [7]. Butyrate, the most biologically active SCFA, is a saturated fatty acid derived from intestinal microbial fermentation of dietary fiber. It performs essential anti-inflammatory and anti-tumor functions through several pathways, in addition to regulating the intestinal flora and maintaining intestinal homeostasis [8, 9]. Butyrate has been shown to activate pyruvate kinase M2 to reprogram the metabolism of colorectal cancer cells and thus suppress their proliferation in an in vitro experiment [10]. Previous breast cancer study showed that butyrate interfered with the glycolysis of cancer cell to increase apoptosis, attributing to suppression of breast cancer development [11]. Increased apoptosis involving overexpression of p53 and p73 in CC cells has also been observed after exposure to butyrate [12]. Butyrate has also been found to inhibit telomerase and release more Cytochrome C (Cytc) and apoptosis-inducing factor (AIF) from mitochondria in CC cells [13]. However, the exact mechanism for butyrate to increase apoptosis in CC cells has not been clear.

As is known, the mitochondrial complex Ι plays a significant role in cell respiration and redox [14], whose dysfunction causes alteration of the mitochondrial membrane potential to increase the release of reactive oxygen species (ROS) from the electron transport chain [15]. Indeed, the levels of NADH and NAD + induced by complex Ι inhibition has been documented to initiate mitochondria-dependent apoptosis [16, 17]. Butyrate, as described above, has been shown to regulate metabolism and accelerate apoptosis in cancer cells [18]. Therefore, we hypothesized that butyrate could inhibit the mitochondria complex Ι to initiate mitochondria-dependent apoptosis in CC cells.

This study aimed to evaluate the inhibitory effects of butyrate on biological features, including cell proliferation, invasion and migration of cervical cells, and to investigate possible mechanisms. Our work found butyrate-induced mitochondria-dependent apoptosis in CC cells, and inhibition of the mitochondrial complex Ι was the underlying mechanism. The findings of the current study will lay a solid foundation for the clinical use of butyrate in the treatment of CC.

## Materials and methods

### Reagents, chemicals and kits

Sodium butyrate (purity > 98%, E2022019) was purchased from Shanghai Aladdin Biochemical Technology Co., Ltd. (Shanghai, China). A 100mM butyrate stock solution was prepared, filtered with a 0.22µM filter membrane and stored at -20 °C. Primary antibodies to glyceraldehyde-3-phosphate dehydrogenase (GAPDH, AB-P-R001) and Caspase 3 (AC0301) were from Hangzhou Goodhere Biotechnology co., LTD and Beyotime Biotechnology (Shanghai, China), respectively. The primary antibodies tp cleaved-Caspase 3 (9661 S), Cyclin D1 (E3P5S), Caspase 9 (#9508) and B-cell lymphoma 2 (Bcl-2) associated X (Bax, 2772) were all purchased from Cell Signaling Technology, Inc. (Shanghai, China). Caspase 8 (ET1612-70), Caspase 12 (HA500144), Cytc (R1510-41), cleaved-Caspase 9 (ER60008), apoptotic protease activating factor 1 (Apaf-1, R1312-20) and Bcl-2 (ER1706-47) were obtained from Hangzhou HuaAn Biochemical Technology Co., Ltd. (Hangzhou, China). Total OXPHOS Rodent WB Antibody Cocktail (MS604) was from Abcam Trading Co., Ltd (Shanghai, China). Poly (ADP-ribose) polymerase (PARP) /cleaved-PARP (WL01932) was from Wanlei Biotechnology Co., Ltd (Shenyang, China). The NAD+/NADH Assay Kit (S0175) was purchased from Beyotime Biotechnology Co., Ltd (Shanghai, China). These primary antibodies were diluted 1:1000 and stored at 4 °C. The secondary antibodies were used at a concentration of 1:5000 and purchased from Proteintech Group, Inc. (Wuhan, China).

### Cell culture

The human CC cell line HeLa and Ca Ski were from the American Type Culture Collection (ATCC, Rockville, USA). HeLa and Ca Ski cell lines were grown in DMEM and RPMI 1640, respectively, with 10% fetal bovine serum (FBS) and incubated at 37 °C with 5% CO_2_.

### CCK-8 proliferation assay

Cell proliferation was assessed with a cell counting kit (CCK-8, CK04, Dojindo, Shanghai, China). CC cells were seeded in a total number of 3 × 10^3^ cells in 96-well plates 24 h before to the start of the experiment. The cells were then incubated with 5mM butyrate for 12, 24, 48 and 72 h. One hundred microliters of 10% CCK-8 solution was added to each microwell and the microplates were further incubated for 2 h. The supernatant was collected and the absorbance was measured at 450 nm by the Microplate reader (ThermoScientific, VarioskanLUX).

### EdU staining test

The Cell-Light EdU Apollo567 In Vitro Kit (C10310-1, Ribobio, Guangzhou, China) was obtained to detect DNA replication in cancer cells. Monolayers of HeLa and Ca Ski cells pre-grown in 12-well microplates were incubated for 24 h and then treated with 5mM butyrate for 48 h, followed by EdU labeling, cell immobilization, Apollo staining and DNA staining. The red fluorescence intensities were imaged using a fluorescence microscope (TOKYO, CKX3-SLP).

### Flow cytometry for apoptosis and cell cycle analysis

The FITC Annexin V Apoptosis Detection Kit (556,547, Becton Dickinson, Shanghai, China) was employed to detect apoptosis and cell cycle of CC cells. Human CC cells were seeded in 6-well plates and treated with 5mM butyrate for 48 h. Cells for apoptosis detection were collected after centrifugation and resuspended in 500µL binding buffer with 5µL of Annexin V and 5µL of propidium iodide (PI), and incubated in the dark for 15 min at room temperature. But for cell cycle assay, the collected cells were fixed with 75% ethanol overnight and then stained with PI. Flow cytometer (Becton Dickinson, FACSCantoII) was used for apoptosis and cell cycle analysis.

### Wound healing assay

HeLa and Ca Ski cells were seeded into 6-well plates and grown to ~ 100%. A perpendicular line to the same location in each well was made with a pipette tip. 2% FBS culture medium containing 5mM butyrate was added, incubated at 37 °C, 5% CO_2_ and imaged at 0, 12, 24, 48 h using a light microscope (Nikon, CI-L).

### Transwell Assay

200µL of basement membrane (Corning Life Science, Shanghai, China) diluted with phosphate buffer saline (PBS) were added to the upper chambers and incubated at 37 °C, 5% CO_2_ for 2 h to discard unnecessary PBS. The upper chambers for migration did not require any treatment. HeLa and Ca Ski cells were collected and resuspended in serum-free medium and then inoculated into the upper chambers. The lower chambers were filled with culture medium containing 10% FBS. The chambers of HeLa and Ca Ski cells were incubated at 37 °C, 5% CO_2_ for 8 and 17 h, respectively, then fixed with 4% formalin for 30 min, stained with 0.1% crystal violet for 15 min and finally observed in light microscope (Nikon, CI-L).

### Western blot analysis

CC cells were seeded in 6-well plates and collected after treatment with butyrate for 48 h. RIPA lysis buffer (Beyotime Biotechnology, Shanghai, China) and protease inhibitor PMSF (ThermoFisher, Shanghai, China) were used for protein extraction. Concentrations of extracted proteins were determined by BCA assay kits (Beyotime Biotechnology, Shanghai, China). Protein samples were separated by sodium dodecyl sulfate-polyacrylamide gelelectrophoresis (SDS-PAGE) and transferred to polyvinylidene fluoride (PVDF) membranes (Bio-Rad Laboratories, Inc. Shanghai, China). The membrane was blocked with 5% skim milk at room temperature for 1.5 h and then incubated with primary antibodies overnight at 4 °C on a shaker. Membranes were washed three times with Tris-buffered saline with Tween (TBST) and incubated with the secondary antibody for 2 h at room temperature. The band was scanned using an Amersham Image 680 imaging system and quantified by densitometry using Image J software (National Institutes of Health, Bethesda, MD). Relative expressions of proteins were calculated using GAPDH as an internal control.

### TUNEL apoptosis analysis

The TUNEL apoptosis assay kit (40307ES20, Yeasen Biotechnology Co, Ltd. Shanghai, China) was used to examine apoptosis of human CC cells. Human CC cells were seeded in 12-well plates containing climbing sheets for 24 h. The experimental group was exposed to 5mM butyrate for 48 h and then washed three times with PBS. Treated cells were fixed with 4% paraformaldehyde for 30 min, resuspended in PBS with 0.3% Triton X-100 and incubated at room temperature for 5 min. TUNEL solution (50µL) was added to the sample, followed by incubation at 37 °C for 60 min in the dark. Antifading mounting medium (with DAPI, Solarbio Science & Technology Co., LTD. Beijing, China) was used for sealing and the stained CC cells were examined with a fluorescence microscope (Becton Dickinson, FACSCantoII).

### ROS production assay

The experimental group treated with 5mM butyrate for 48 h: Mitochondrial ROS was qualitatively determined by a superoxide fluorescent probe (40778ES50, Yeasen Biotechnology Co, Ltd. Shanghai, China). The experimental group treated with 0.22µM rotenone for 48 h: ROS was qualitatively determined by the ROS Assay Kit (S0033S, Beyotime Biotechnology Co, Ltd. Shanghai, China). HeLa cells were cultured in 12-well plates containing climbing sheets for 24 h. The experimental group was treated with butyrate for 48 h, followed by three washes with PBS. ROS working solution, an ethidium bromide (EB) derivative that can be rapidly oxidized by ROS, was added and the plates were incubated at 37 °C in the dark for 10 min. The sealed samples were examined with the fluorescence microscope (TOKYO, CKX3-SLP).

Flow cytometry was also employed for qualitative detection of ROS. HeLa cells were seeded in 6-well plates and treated with butyrate/rotenone for 48 h. After treatment, ROS working solution was added and incubated in the dark for 10 min at 37 °C. The ROS expression was detected by flow cytometer (Becton Dickinson, FACSCantoII).

### Cytc expression assay

HeLa cells were seeded in 12-well plates containing climbing sheets and exposed to 5mM butyrate for 48 h. After three washes with PBS, cells were fixed with 4% paraformaldehyde for 15 min, washed again in PBS, treated with 0.1% Triton X-100 for 5 min, blocked with 1% bovine serum albumin for 30 min and incubated with Cytc primary antibodies (1:500) overnight in the dark at 4 °C. The treated cells were then washed with PBS and incubated with the secondary antibody (ThermoFisher, Shanghai, China) for 2 h in the dark at room temperature. Fluorescence microscope (Becton Dickinson, FACSCantoII) were used for sample detection.

### Real-time q-PCR

Gene expression of the mitochondrial complex Ι was determined by real-time q-PCR (RT-qPCR). Total RNA was extracted from HeLa cells, GAPDH was used as an internal control. Fold changes were calculated by the 2^−∆∆Ct^ method (Primer information is shown in the Supplementary table).

### Energy targeted metabolomics sample preparation

HeLa cells were seeded in 10 cm dishes and exposed to 5mM butyrate for 48 h. Cells were grown to approximately 90% confluency and then harvested. After washing with cold PBS and cold normal saline three times, the cells were dissolved in 1ml of cold 4/4/2 (v/v/v) methanol/acetonitrile/water. Then, all cell samples were collected in 1.5ml centrifuge tubes stored in a refrigerator at − 80 °C after flash freezing in liquid nitrogen. Samples were separated by the Agilent 1,290 Infinity UPLC system at Shanghai Applied Protein Technology Co.,Ltd. (Shanghai, China). Data were acquired using QTRAP5500 (ABSCIEX) mass spectrometry in negative ionization mode. Chromatographic peak area and retention time were extracted by Multiquanta software. Retention time corrected for energy metabolite standard.

### Levels of mitochondrial NADH and NAD + assay

NADH and NAD + concentrations were quantitatively determined by the NAD+/NADH Assay Kit (Beyotime Biotechnology, Shanghai, China). HeLa cells were seeded in 6-well plates and treated with butyrate or rotenone for 48 h. Cells were washed twice with PBS, added to NADH and NAD + extract for lysis cell. The Cell suspension was obtained by centrifugation at 1,2000 g for 5 min at 4 °C. Plot the standard curve and measure the absorbance samples at 450 nm.

### Flow cytometry for mitochondrial membrane potential analysis

Mitochondrial membrane potential was detected by JC-1 fluorescent dye (Nanjing Jiancheng Bioengineering institute, Nanjing, China). HeLa cells were seeded in 6-well plates and treated with butyrate for 48 h. Cells were washed twice with PBS and suspended in JC-1 working solution. Then, cells were incubated at 37 °C, 5% CO_2_ for 20 min, rewashed in incubation buffer, resuspended with incubation buffer. Green fluorescence was detected through the FITC channel and red fluorescence was detected through the PE channel by flow cytometry (Becton Dickinson, FACSCantoII).

### Rotenone IC_50_ assay

Detected tolerance of HeLa cells to rotenone concentration with a cell counting kit (CCK-8, CK04, Dojindo, Shanghai, China). 3000 cells were added to 96-well plates and cultured for 24 h. Cells were then incubated with 0.1µM, 0.25µM, 0.5µM, 0.75µM, 1µM, 2µM rotenone for 48 h. One hundred microliters of 10% CCK-8 solution were added to each microwell and the microplates were further incubated for 2 h. The supernatant was collected and the absorbance was measured at 450 nm.

### Statistical analysis

Data are presented as mean ± standard deviation (SD). Differences between two groups were assessed using the two-tailed unpaired Student’s t-test, with a P-value less than 0.05 considered statistically significant.

## Results

### Butyrate suppressed CC cells proliferation

**Fig. 1:**
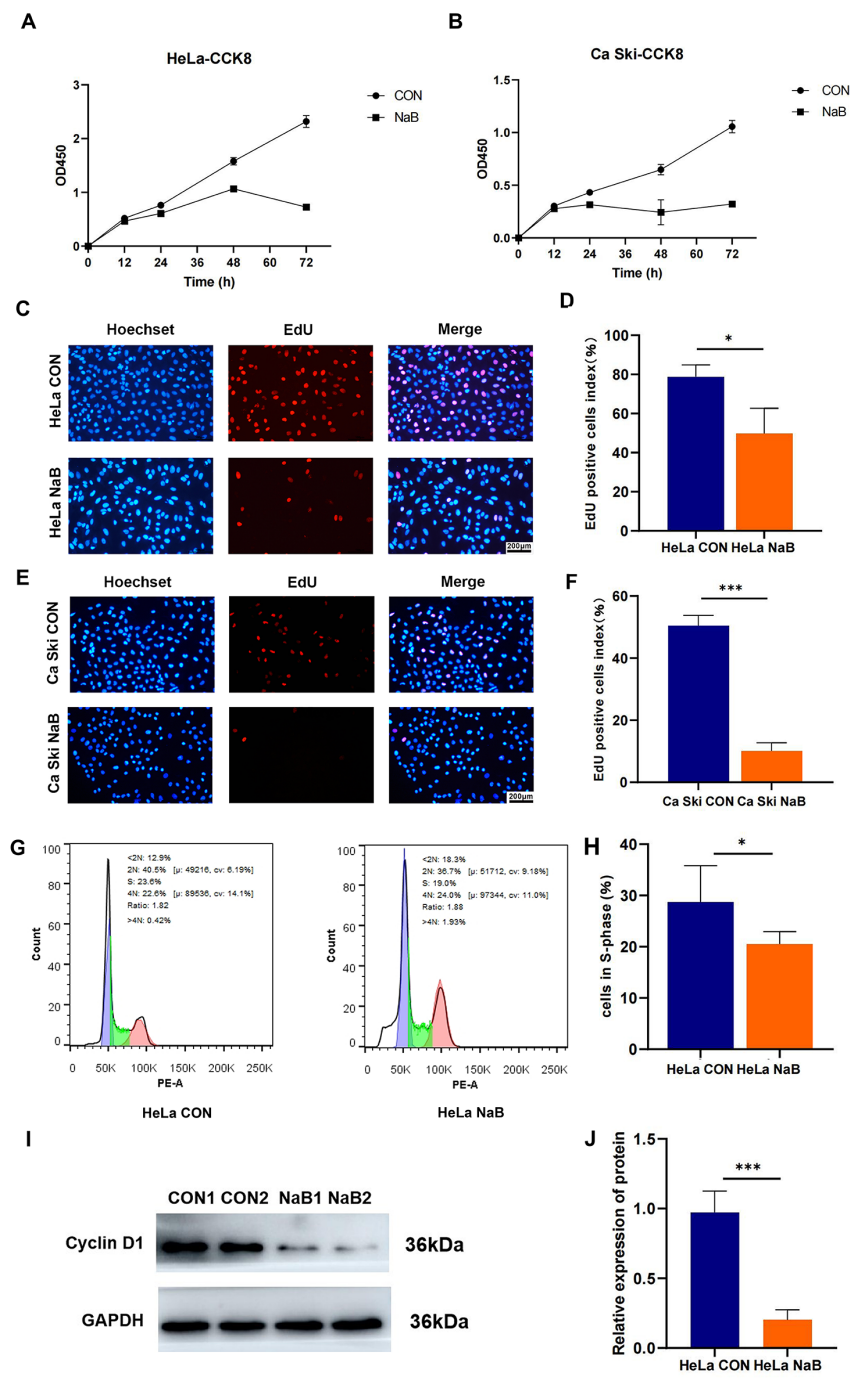
Butyrate suppressed the proliferation of human CC cells. CCK8 was used to determinate the proliferation of **(A)** HeLa and **(B)** Ca Ski cells treated with 5mM butyrate for 12, 24, 48 and 72 h. The EdU staining assay was performed to detect apoptotic cells in **(C)** HeLa and **(E)** Ca Ski cells. (**D, F)** Quantitative analysis of the proportion of red fluorescence in EdU. **(G)** The cell cycle distribution of HeLa cells was analyzed by flow cytometry. **(H)** Quantitative analysis of S-phase in HeLa cells. **(I)** Western blot analysis of Cyclin D1. **(J)** Quantitative analysis of Western blot in CON and NaB groups. CON: Control group; NaB: Group treated with 5mM butyrate for 48 h. All data above are expressed as mean ± SD. * *P* < 0.05; *** *P* < 0.001

Human CC cells HeLa and Ca Ski were treated with 5mM butyrate for 12, 24, 48, 72 h to observe the effect of butyrate on cell proliferation using CCK-8 detection. The results showed an inhibition of cell proliferation for HeLa and Ca Ski cells at different time points (Fig. 1A, B). After 48 h of butyrate treatment, the curve started to decrease significantly compared to the CON group. EdU staining also showed that, compared to the control group, the red fluorescence intensity of HeLa cells and Ca Ski cells decreased by about 30% and 40% after 48 h of exposure to butyrate (Fig. 1C, E), indicating that butyrate significantly inhibited cell growth of HeLa (*P* = 0.0238) and Ca Ski cells (*P* < 0.0001) (Fig. 1D, F). Cell cycle distribution analysis indicated that the number of cells in S-phase was reduced from 23 to 19% after butyrate treatment in HeLa cells (Fig. 1G) and had a statistical difference (*P* = 0.0416) (Fig. 1H). Western blot results showed that of Cyclin D1 protein expression was decreased by butyrate treatment (Fig. 1I, J), implying that butyrate suppressed cell proliferation in CC cells by arresting the cell cycle in the G1 phase.

### Butyrate reduced the invasion and migration of CC cells

**Fig. 2:**
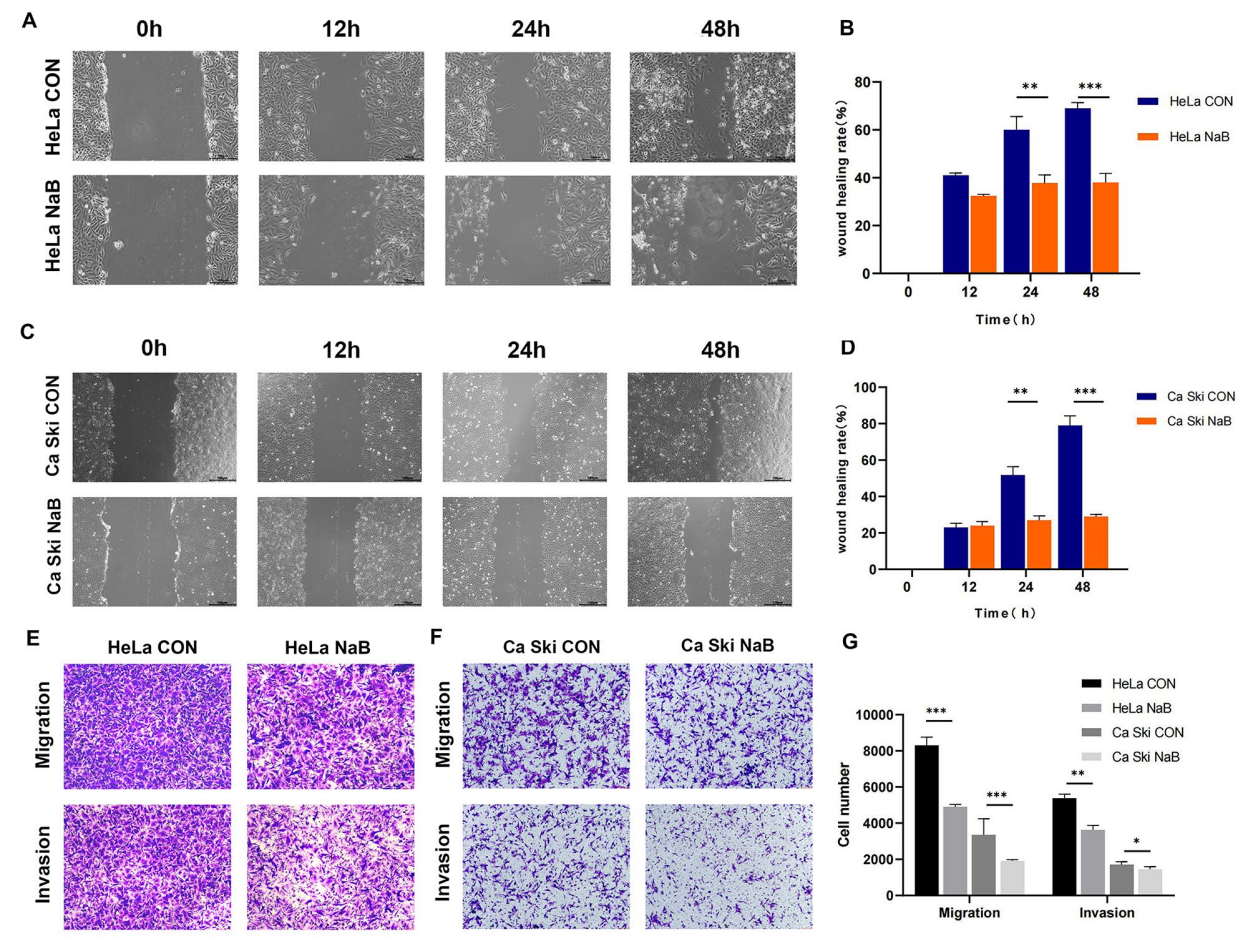
Butyrate inhibited the invasion and migration ability of CC cells. The wound healing experiment was used to determine the migration of **(A)** HeLa and **(C)** Ca Ski cells treated with 5mM butyrate for 12, 24, 48 h. **(B, D)** Quantitative analysis of wound healing rate. **(E, F)** The effects of butyrate on CC cell invasion and migration were using the transwell assay. **(G)** Quantitative analysis of the transwell in the CON and NaB groups. CON: Control group. NaB: Group treated with 5mM butyrate for 48 h. Data were expressed as mean ± SD. * *P* < 0.05; ** *P* < 0.01; *** *P* < 0.001

Because invasion and migration are the important CC phenotype, we further evaluated the effects of butyrate on these aspects in the study. The results of the Wound healing experiment of HeLa and Ca Ski cells showed that butyrate suppressed cell migration (Fig. 2A, C), especially at 48 h (HeLa cells *P* = 0.0003, Ca Ski cells *P* < 0.0001) (Fig. 2B, D). Furthermore, transwell experiments demonstrated that butyrate treatment decreased the number of stained cells (Fig. 2E, F), with statistical significance (migration of HeLa cells P < 0.0001, invasion of HeLa cells *P* < 0.0001, migration of Ca Ski cells *P* = 0.0071, invasion of Ca Ski cells *P* = 0.0377) (Fig. 2G), revealing that butyrate remarkably inhibits cell invasion and migration in HeLa and Ca Ski cells.

### Butyrate promoted apoptosis of CC cells

**Fig. 3:**
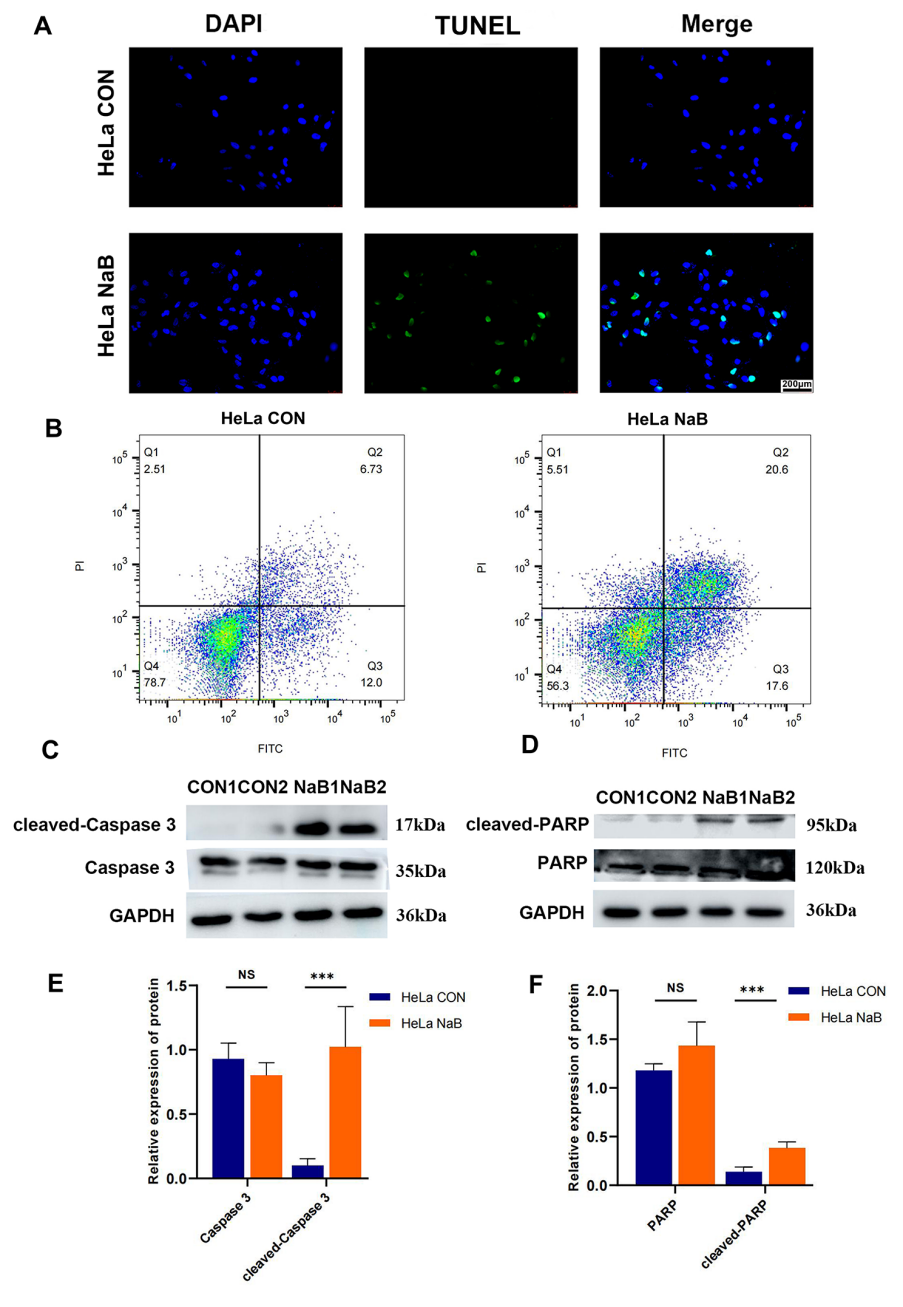
Butyrate promoted apoptosis of CC cells. **(A)** TUNEL and **(B)** Annexin V/PI staining were used to qualitatively and quantitatively examine apoptosis of HeLa cells. **(C, D)** Western blot analysis of Caspase 3, cleaved-Caspase 3, PARP, and cleaved-PARP. **(E, F)** Quantitative analysis of Western blot in the CON and NaB groups. CON: Control group. NaB: Group treated with 5mM butyrate for 48 h. Data were expressed as means ± SD. *** *P* < 0.001

The of TUNEL staining results demonstrated that the green fluorescence intensity of butyrate-treated HeLa was significantly increased, indicating that butyrate exactly increased apoptosis of HeLa cells (Fig. 3A). Annexin V /PI staining and flow cytometry were further employed to quantify apoptosis of HeLa cells. The rate of early (Annexin V +, PI -) and late (Annexin V +, PI +) apoptosis rate of HeLa cells was 22.6% and 22.7% in the butyrate group, only 12.1% and 6.1% in the control group (Fig. 3B). We further investigated the molecular changes underlying the inhibitory effect of butyrate on HeLa cells. Western blot analysis was conducted to detect the levels of cleaved-Caspase 3 and cleaved-PARP, the main drivers of apoptosis, and we observed a significant increase in the expression of these proteins after treatment with butyrate (Fig. 3C-F).

### Butyrate activated mitochondria-dependent apoptosis pathway

**Fig. 4:**
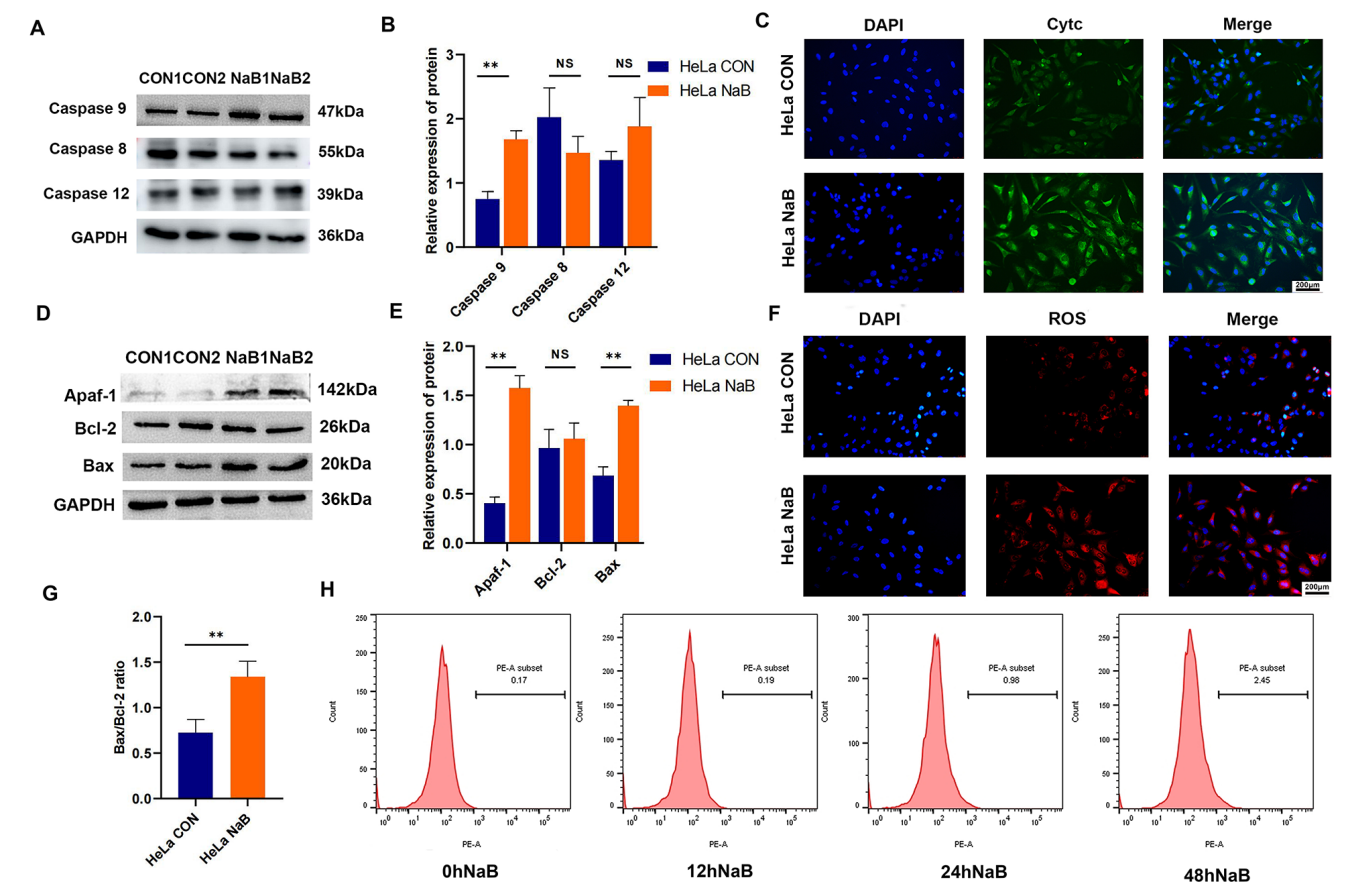
Butyrate activated the mitochondrial-dependent apoptosis pathway in CC cells. Western blot analysis of **(A)** Caspase 8, 9, 12, **(D)** Apaf-1, Bcl-2 and Bax. **(B, E)** Quantitative analysis of Western blot in the CON and NaB groups. **(G)** Comparison of the Bax/Bcl-2 protein ratio between the CON group and the NaB group. **(C)** Cytc immunofluorescence staining in the CON and NaB groups. **(F)** Immunofluorescence for ROS. (H) Flow cytometry for quantitative detection of ROS. CON: Control group. NaB: Group treated with 5mM butyrate for 48 h. Data were expressed as meas ± SD. ** *P* < 0.01

The Western blot results showed that butyrate up-regulated the expression of Caspase 9, but not Caspase 8 or 12 (Fig. 4A, B), indicating that the mitochondria-dependent pathway is the response to butyrate’s inhibition effects on HeLa cells. Moreover, remarkable elevation of Apaf-1 and Cytc was found after exposure to butyrate using Western blot and fluorescence staining (Fig. 4C-E). Although the expression of the pro-apoptotic protein Bax increased, but the anti-apoptotic protein Bcl-2 remained unchanged (Fig. 4D, E), the Bax/Bcl-2 ratio increased after butyrate treatment (*P* = 0.0017) (Fig. 4G). ROS assessment using fluorescence staining showed an increase in red fluorescence intensity in HeLa cells with butyrate treatment (Fig. 4F). Likewise, flow cytometry detection showed that the red peak in the butyrate treatment group shifted to the right by about 2.28% at 48 h, suggesting that butyrate increased intracellular ROS production in HeLa cells (Fig. 4H).

### GPR may not be the key mechanism of butyrate’s anti-CC effect

**Fig. 5:**
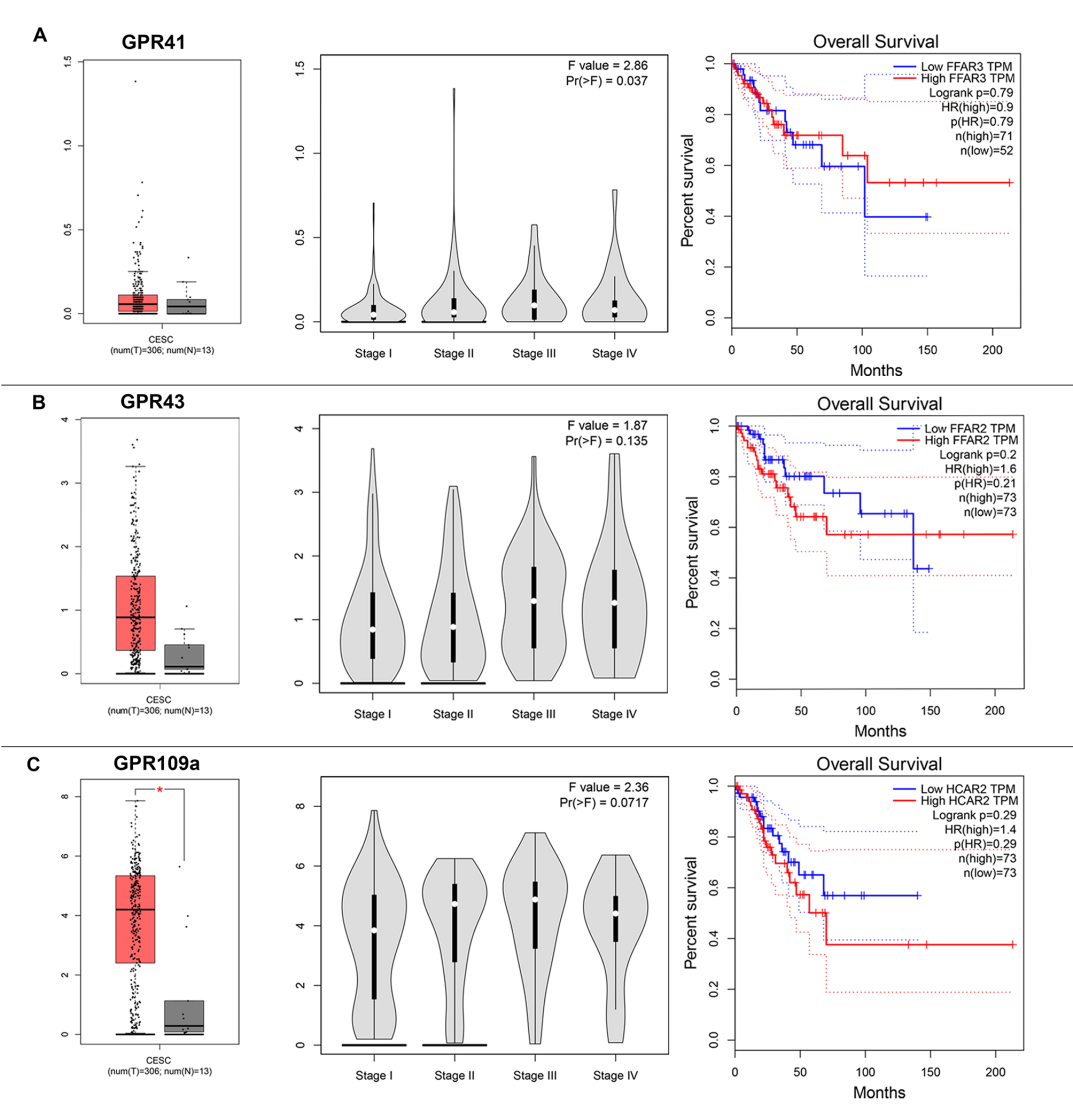
GPR may not be the key mechanism of butyrate’s anti-CC effect. TCGA data for GPR41 **(A)**, GPR43 **(B)**, GPR109a **(C)** in human CC tissue and adjacent normal tissue, and at different stages of human CC. The prognosis of patients with CC with low or high expression of GPR41 **(A)**, 43 **(B)**, 109a **(C)**

The TCGA data showed that there was a statistical difference in the expression of G protein-coupled receptor 109a (GPR109a) between human CC tissue and normal tissues (Fig. 5C), but insignificant difference for GPR41 (Fig. 5A), GPR43 (Fig. 5B). And there was no statistical difference in the expression of GPR41, 43 and 109a when the CC stage developed. In addition, we re-analyzed TCGA data for correlation between butyrate receptors and prognosis in patients with CC. The prognosis of patients with CC was not changed when patients expressed high or low GPR41, 43, 109a. These results suggested that butyrate receptors GPR41, 43, 109a may not be major contributors to the anti-tumor effects of butyrate in CC.

### Butyrate reduced the levels of mitochondrial NADH and NAD + in CC cells

**Fig. 6:**
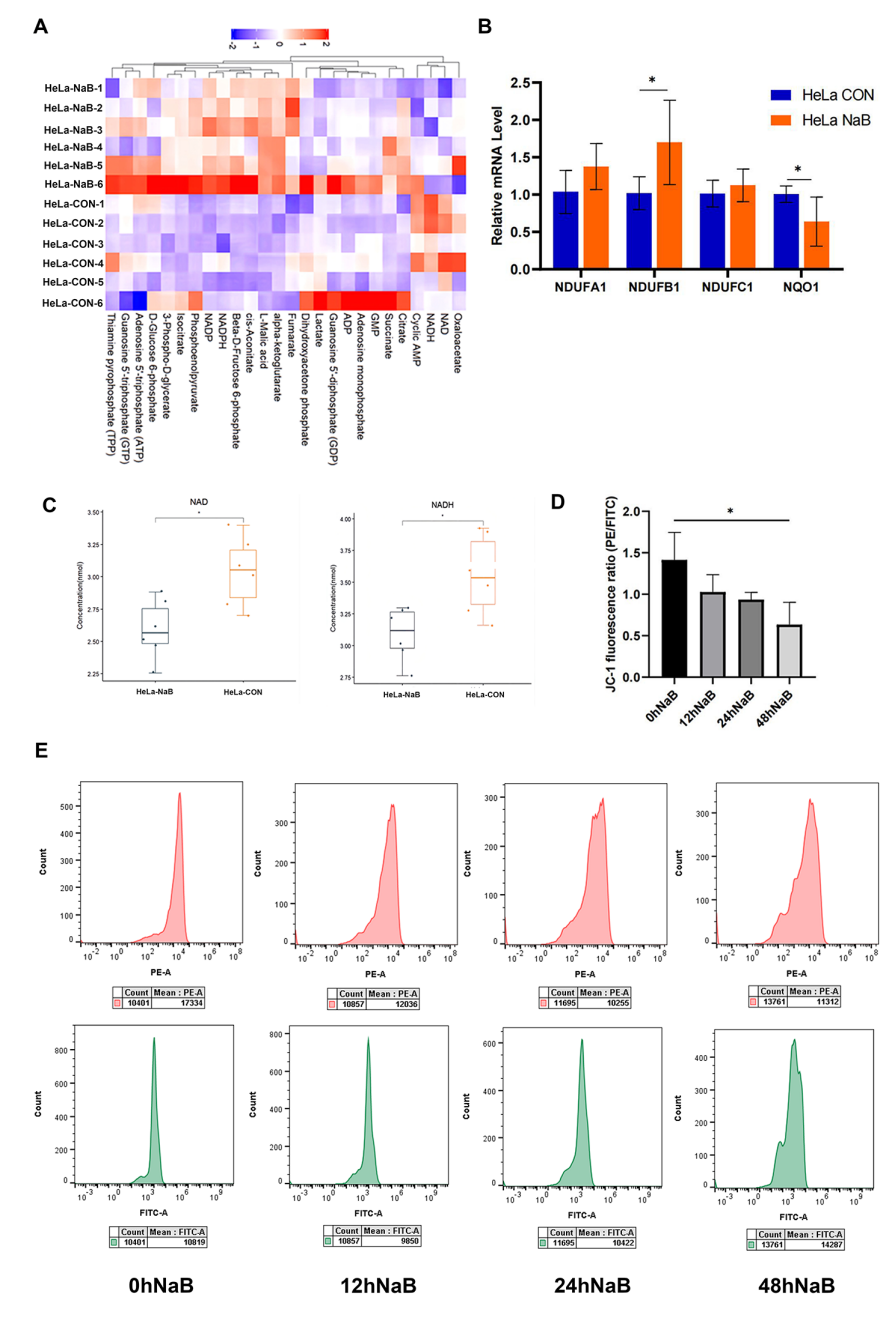
Butyrate-induced mitochondrial-dependent apoptosis by inhibition of the mitochondrial complex Ι in CC cells. **(A)** Heat map analysis of changes in energy metabolism between the CON group and the NaB group. **(C)** Levels of mitochondrial NADH and NAD + in CC cells. **(B)** RT-qPCR analysis of related mRNA expression in the CON group and the NaB group. **(E)** Detection of mitochondrial membrane potential by JC-1 flow cytometry. **(D)** Quantitative analysis of the JC-1 fluorescence ratio in HeLa cells treated with 5mM butyrate for 12, 24 and 48 h. CON: Control group. NaB: Group treated with 5mM butyrate for 48 h. Data were expressed as the mean ± SD. * *P* < 0.05

We detected mitochondrial energy metabolism in HeLa cells (Fig. 6A), and found that levels of mitochondrial NADH and NAD + decreased significantly after butyrate treatment (Fig. 6C). Furthermore, the mRNA level of NDUFA1 (*P* = 0.4248), NDUFB1 (*P* = 0.0230), NDUFC1 (*P* = 0.3746) increased compensatively, while NQO1 decreased (*P* = 0.0299), suggesting damage by oxidative stress (Fig. 6B). Mitochondrial membrane potential was detected by flow cytometry, in which a decrease in red fluorescent intensity and an increase in green fluorescent intensity were observed (Fig. 6E). Thus, the mitochondria JC-1 fluorescence rate was significantly reduced at 48 h (*P* = 0.0341) (Fig. 6D), indicating that the mitochondrial membrane potential of HeLa cells decreased after exposure to butyrate.

### Butyrate-induced mitochondrial-dependent apoptosis by inhibition of the mitochondrial complex Ι in CC cells

**Fig. 7:**
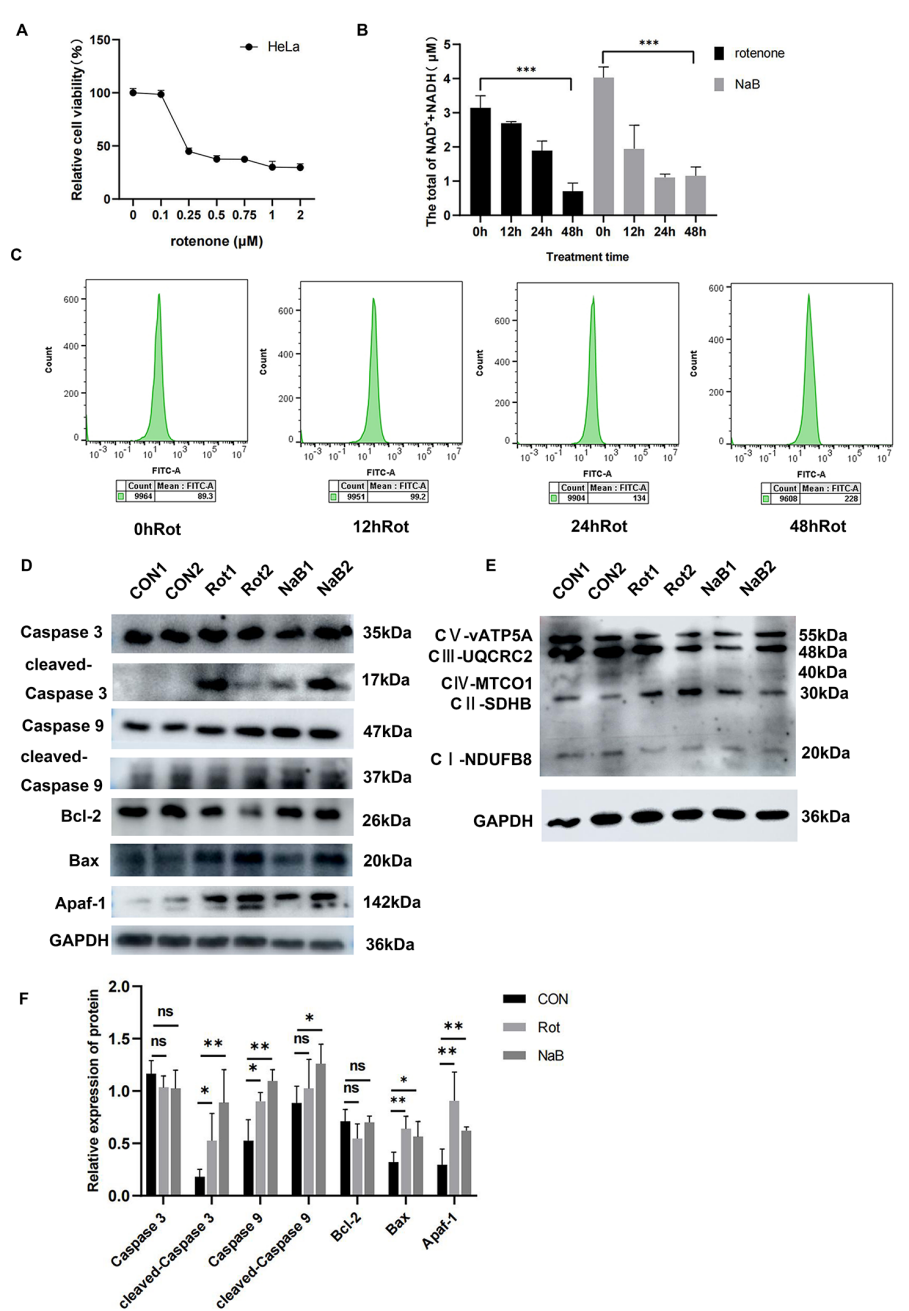
Butyrate inhibited the mitochondrial complex Ι in CC cells. **(A)** CCK8 was used to determine the viability of HeLa cells treated with various concentrations of rotenone for 48 h. **(B)** Levels of mitochondrial NADH and NAD + treated with rotenone or butyrate for 48 h. **(C)** Flow cytometry for quantitative detection of ROS after rotenone treatment. **(D)** Western blot analysis for Caspase 3, cleaved-Caspase 3, Bax, Bcl-2, Caspase 9, cleaved-Caspase 9, Apaf-1 and **(E)** for mitochondrial complex Ι. **(F)** Quantitative analysis of Western blot in CON, Rot and NaB groups. CON: Control group. NaB: Group treated with 5mM butyrate for 48 h. Rot: Group treated with 0.2µM rotenone for 48 h. Data were expressed as mean ± SD. * *P* < 0.05; ** *P* < 0.01; *** *P* < 0.001

As an inhibitor of the mitochondrial complex Ι, rotenone was observed to inhibit CC cell proliferation in a concentration-dependent manner, and we selected 0.25µM as the best treatment concentration (Fig. 7A). The flow cytometry result showed that the green fluorescence intensity of HeLa cells with rotenone treatment increased from 89.3 to 228, suggesting that ROS accumulated after rotenone treatment (Fig. 7C). Butyrate reduced NADH and NAD + levels, with significant statistical difference at 48 h (butyrate *P* = 0.0003, rotenone *P* = 0.0006) (Fig. 7B). Consistent with the effects of rotenone, butyrate also decreased the expression of the mitochondrial complex Ι (Fig. 7E). It is noted that both butyrate and rotenone induced mitochondria-dependent apoptosis in HeLa cells (Fig. 7D, F).

## Discussion

The current study found that butyrate dramatically suppressed tumor cell proliferation, migration, and invasion in CC cell lines through increased apoptosis. The TCGA data revealed that SCFAs-specific receptors may not be the key factor for butyrate-induced apoptosis of CC cells. Mechanistically, our findings originally showed that butyrate inhibited mitochondrial complex I, inducing endogenous mitochondria-mediated apoptosis.

Butyrate has been identified as an anti-tumor chemical, capable of inhibiting the proliferation of tumor cells, including CC cells in vitro [19–22]. Our results further revealed that the growth of HeLa cells was stopped in G1 phase by butyrate. Moreover, the transwell experiment demonstrated that butyrate treatment significantly decreased the migration and invasion of CC cells. Given the importance of triggering apoptosis for butyrate to exert its anti-cancer effects [23], we did related investigations and found that butyrate significantly increased Caspase 9 but not Caspase 8 or 12, accompanied by elevated expression of Apaf-1 and Cytc, indicating activated mitochondria-dependent apoptosis, which is consistent with the findings by Tiwari et al. [24] in skin cancer. Several studies have reported that increasing the Bax/Bcl-2 ratio is critical for butyrate to promote mitochondria-dependent apoptosis [25, 26]. Here, our Western blot result also showed an increase in the Bax/Bcl-2 ratio in CC cells after butyrate treatment, although Bcl-2 expression was not down-regulated.

Butyrate as a primary source of energy has been reported to affect the NADH/NAD + ratio in colonocytes [27]. Butyrate combined with sirtuin inhibitors have been found to promote apoptosis of leukemia cells by lowering NAD + levels [28]. Our metabolic analysis showed that the levels of NADH and NAD + in HeLa cells significantly decreased after butyrate treatment. Importantly, the change in NADH and NAD + levels induced by mitochondrial complex I was considered to be the critical onset of mitochondria-dependent apoptosis [29–31], which was confirmed by mitochondrial apoptosis induced by the complex Ι inhibitor, rotenone in HeLa cells. Overall, our results showed that inhibition of mitochondrial complex Ι was the main mechanism for butyrate-induced mitochondria-dependent apoptosis.

Despite the nutritional effect on normal colonic epithelium [32], butyrate acts as a histone deacetylase inhibitor (HDACi) [33] to increase histone acetylation to down-regulate the expression of anti-apoptotic genes such as Bcl-2, Bcl-xl, while up-regulating pro-apoptotic genes including Bax, BAK, thereby inducing cellular apoptosis [34]. Notably, butyrate altered the expression of genes involved in reprogramming cancer metabolism [35]. In our study, significant alterations in the metabolism of HeLa cells, mainly the reduction in the levels of NADH and NAD+, appeared after treatment with butyrate, indicating the inhibition of the mitochondrial complex I. We also found a decrease in the expression of composed proteins of the complex Ι. Thus, in addition to the direct effect on metabolites, butyrate can be used as HDACi to regulate the expression of genes that encode complex components to decrease the levels of NADH and NAD+. Additionally, butyrate can interact with GPR41, GPR43 and GPR109a to suppress expression of anti-apoptotic genes to induce cellular apoptosis in cancer cells [36]. However, our TCGA-based analysis does not support these pathways in human CC cells.

Interestingly, the HeLa and Ca Ski cell lines used in our study are positive for HPV-18 and HPV-16, respectively, suggesting potential butyrate therapy in CC with HPV infection. Although HPV is known as the important etiological factor in CC, emerging evidence has shown that cervical epithelium carcinogenesis also depends on the vaginal microbiota in HPV-positive patients [37]. For HPV-positive woman, bacterial diversity and composition were more complex compared to HPV-negative woman [38–40]. Furthermore, investigations revealed that cervical microbiota such as *Fusobacterium* [41] and *Chlamydia trachomatis* [42, 43] can increase anti-inflammatory cytokines to play an important role in the development of immunosuppressive microenvironment related to HPV infection and persistence. As is known, butyrate is the main metabolite of *Lactobacillus* and *Fusobacterium*, indicating that butyrate may play a critical role for vaginal microbiota against HPV-positive CC. Notably, an early study in 1998 demonstrated the direct suppression effect of butyrate on HPV-11 replication [44].

## Conclusion

In conclusion, our results revealed that butyrate suppressed cell proliferation, invasion and migration of CC cell lineages by reducing the mitochondrial complex I to activate the mitochondria-dependent apoptosis pathway. Given the inhibition effect on HPV positive CC cells, butyrate may have a potential therapeutic effect in HIV-positive CC patients.

## Electronic supplementary material

Below is the link to the electronic supplementary material.

Supplementary Material 1

Supplementary Material 2

## Data Availability

The datasets generated and/or analysed during the current study are available in the [jianguoyun] repository, [https://www.jianguoyun.com/p/DYU8oQUQxdjRCxiKj4gFIAA]
