# Supplementary material for: Butyrate inhibits the mitochondrial complex Ι to mediate mitochondria-dependent apoptosis of cervical cancer cells
Source: BMC Complement Med Ther. 2023 Jun 27;23:212. doi: 10.1186/s12906-023-04043-3 (PMC10304623; doi:10.1186/s12906-023-04043-3)
Supplement: Supplementary file 1 — Supplementary Material 1 [file 12906_2023_4043_MOESM1_ESM.pdf]

Table 1. Primers used for genes of interests for RT-qPCR

Gene Name	Forward Primer (5’-3’)	Reverse Primer (5’-3’)
NDUFA1	ATGTGGTTCGAGATTCTCCCC	CCTGTGGATGTACGCAGTAGC
NDUFB1	GTCCCTATGGGATTTGTCATTGG	CAGTTAGCCGTTCATCACTCTT
NDUFC1	CCTTCAGTGCGATCAAAGTTCT	CAGCCAGTCAGGTTTGGCAT
NQO1	GAAGAGCACTGATCGTACTGGC	GGATACTGAAAGTTCGCAGGG
GAPDH	CAGGGCTGCTTTTAACTCTGGTAA	GGGTGGAATCATATTGGAACATGT
