# Supplementary material for: Butyrate inhibits the mitochondrial complex Ι to mediate mitochondria-dependent apoptosis of cervical cancer cells
Source: BMC Complement Med Ther. 2023 Jun 27;23:212. doi: 10.1186/s12906-023-04043-3 (PMC10304623; doi:10.1186/s12906-023-04043-3)
Supplement: Supplementary file 2 — Supplementary Material 2 [file 12906_2023_4043_MOESM2_ESM.pdf]

Figure 1 supplemental I

Cyclin D1

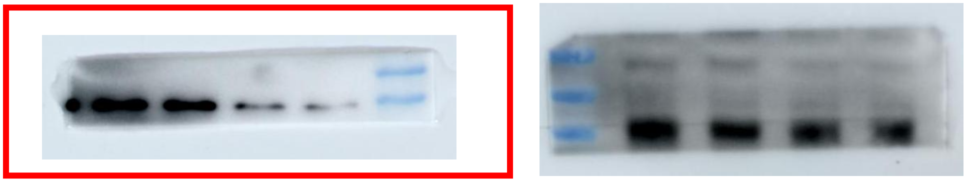

GAPDH

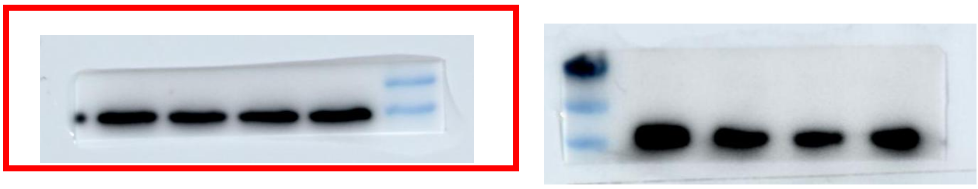

Figure 3 supplemental C, D

PARP + cleaved-PARP

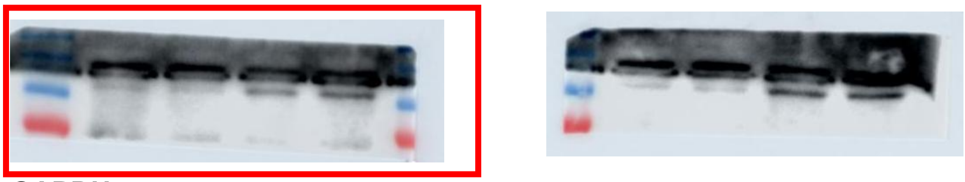

GAPDH

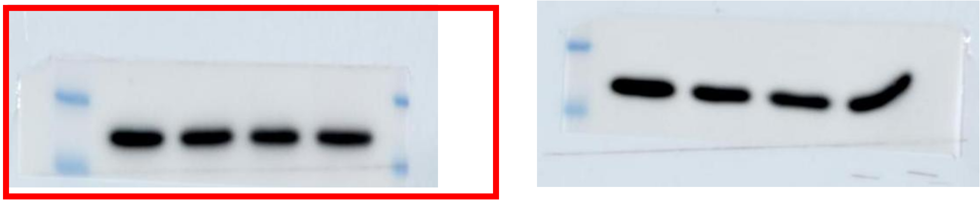

Caspase 3

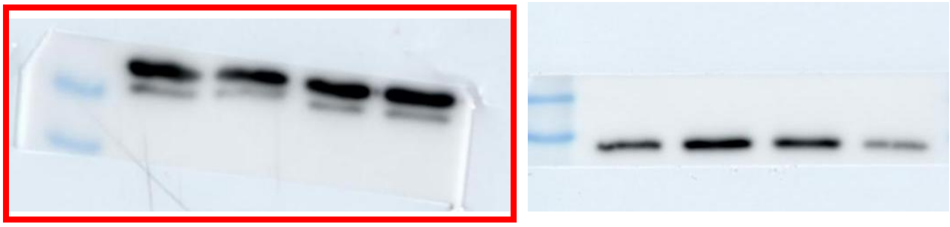

cleaved-Caspase 3

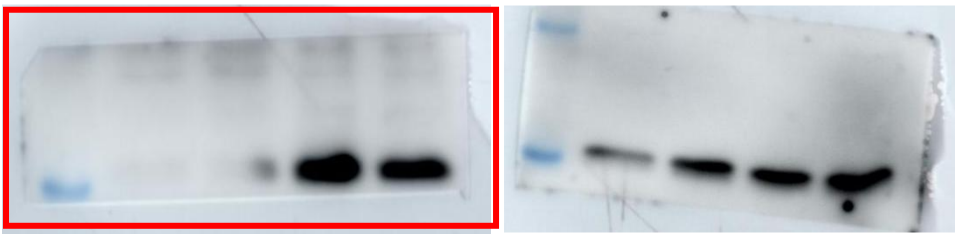

GAPDH

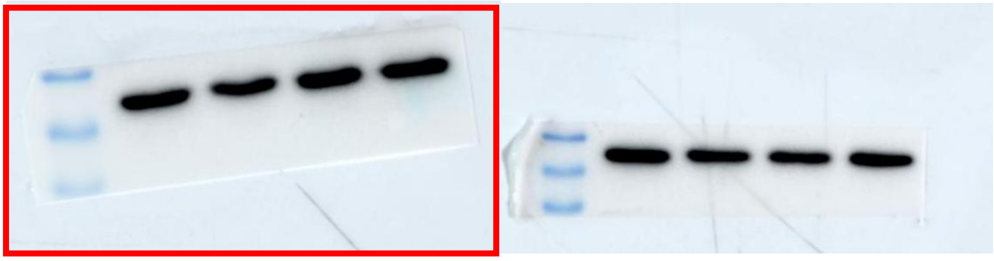

Figure 4 supplemental A, D

Caspase 9

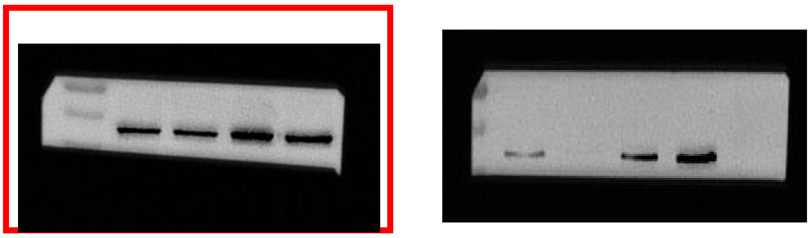

Caspase 8

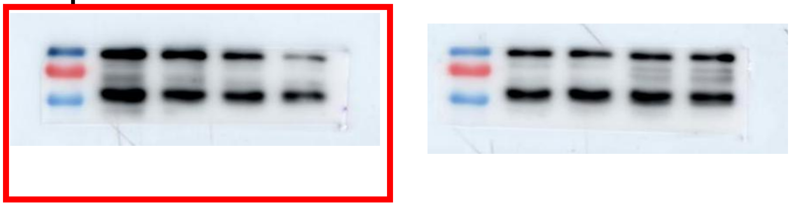

Caspase 12

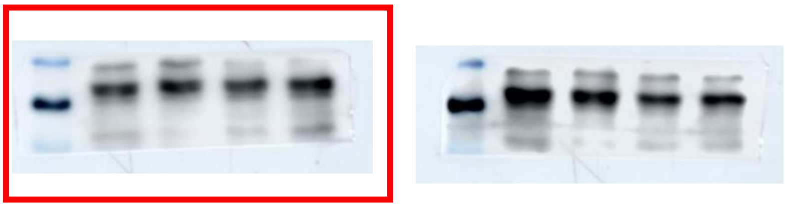

GAPDH

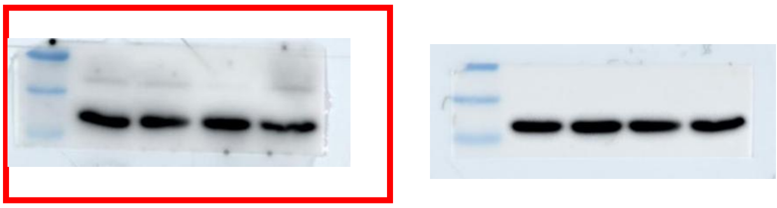

Bax

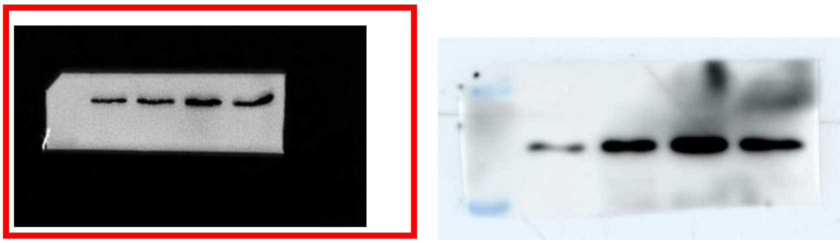

Bcl-2

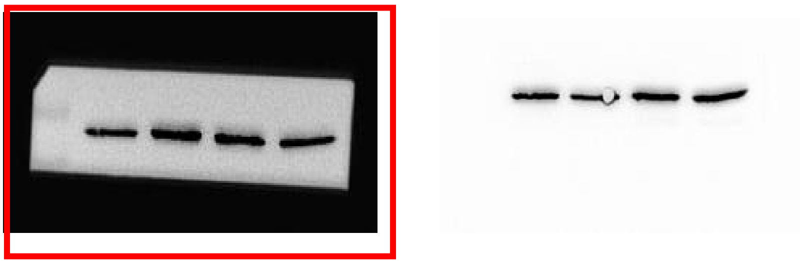

Apaf-1

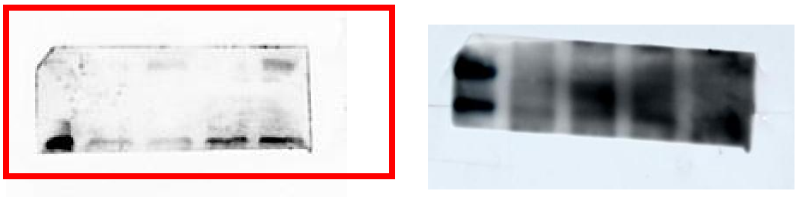

GAPDH

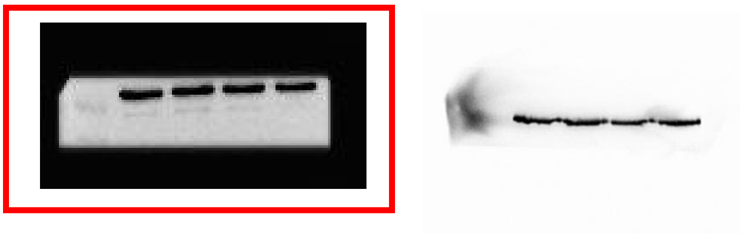

Figure 7 supplemental D, E

Caspase 3

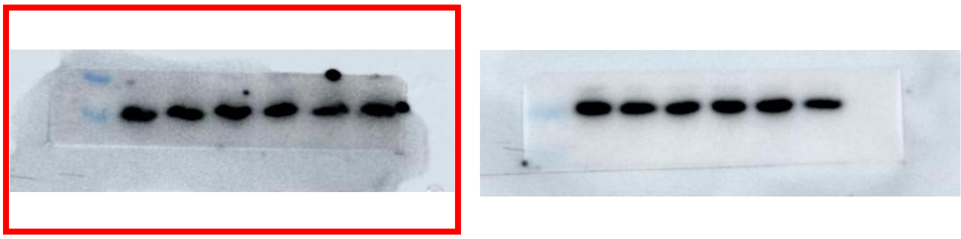

cleaved-Caspase 3

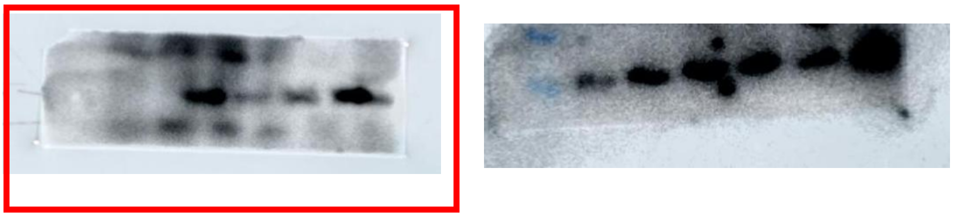

Bcl-2

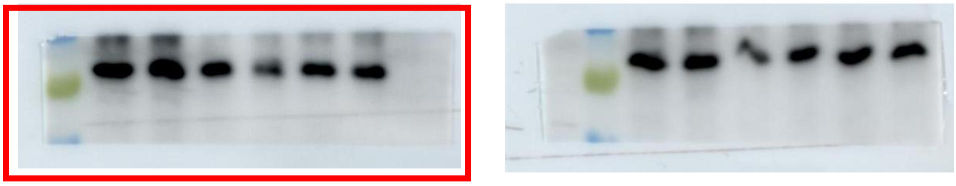

GAPDH

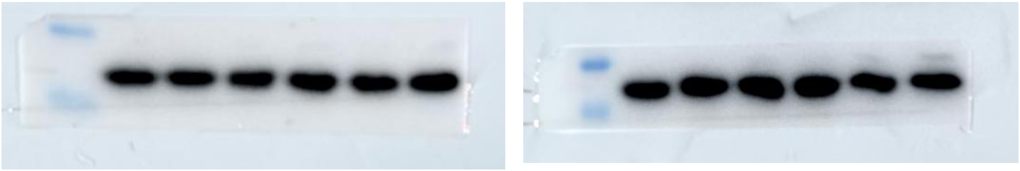

Bax

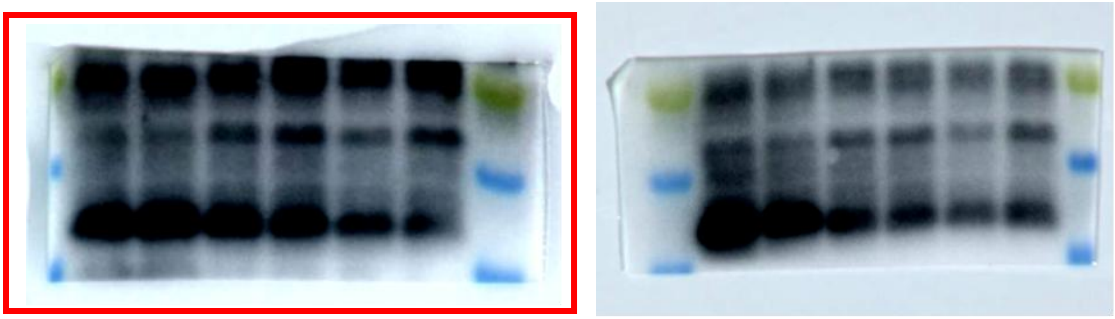

GAPDH：

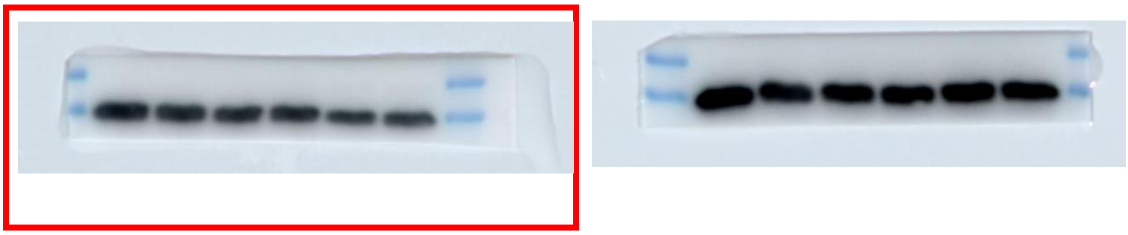

Apaf-1

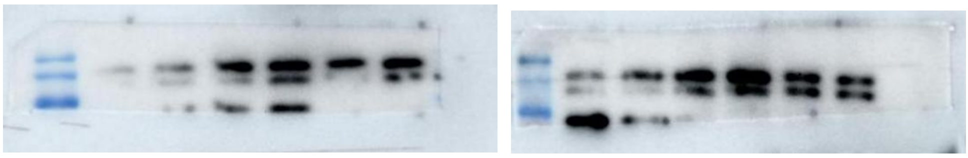

Caspase 9

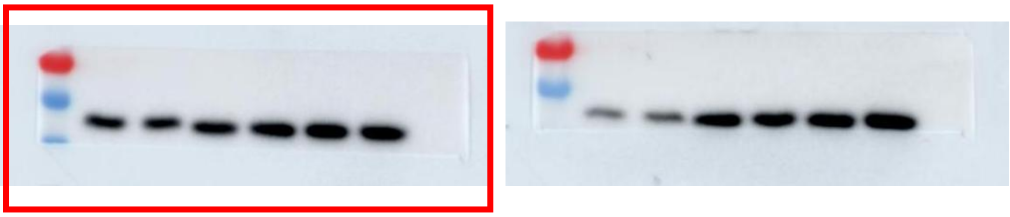

GAPDH

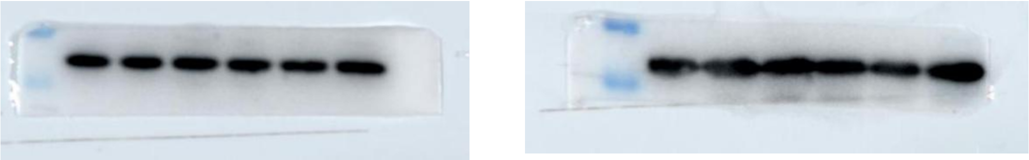

cleaved-Caspase 9

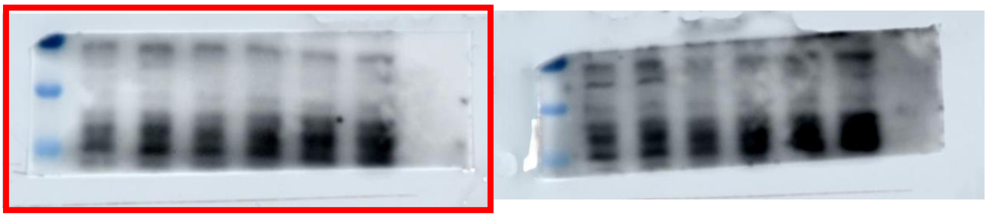

GAPDH

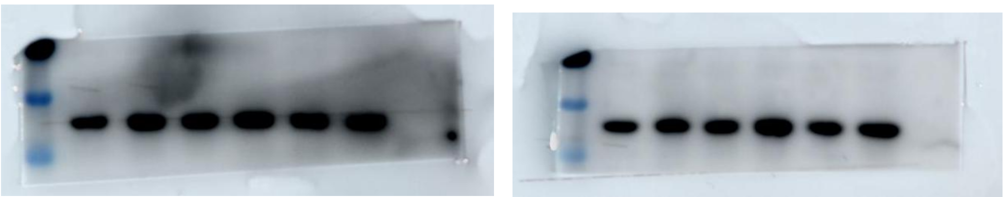

Mitochondrial complex I + GAPDH

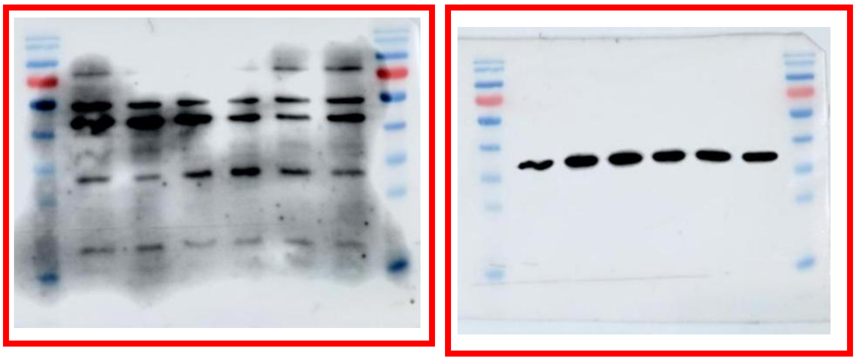

The original, uncropped, and replicated blots were presented. The first column of blots correspond to cropped.
